# Deciphering the Genetic Basis of Congenital Vertebral Malformations Through a Stepwise Diagnostic Approach

**DOI:** 10.3390/ijms27041752

**Published:** 2026-02-11

**Authors:** Anna Szoszkiewicz, Anna Sowińska-Seidler, Aleksandra Wnuk-Kłosińska, Ewelina Bukowska-Olech, Karolina Biel, Karolina Matuszewska, Marcin Biel, Magdalena Badura-Stronka, Renata Glazar, Anna Jakubiuk-Tomaszuk, Maciej Krawczyński, Krzysztof Szczałuba, Karolina Śledzińska, Marzena Wiśniewska, Aleksander Jamsheer

**Affiliations:** 1 Department of Medical Genetics, Poznan University of Medical Sciences, 60-806 Poznan, Poland; 2 Department of Medical Genetics, Doctoral School, Poznan University of Medical Sciences, 61-701 Poznan, Poland; 3 Diagnostyka GENESIS, 60-529 Poznan, Poland; 4 Department of Laboratory Diagnostics, Poznan University of Medical Sciences, 60-569 Poznan, Poland; 5 University Clinical Hospital in Poznań, 60-786 Poznan, Poland; 6 Department of Radiology, HCP Medical Center Poznan, 61-485 Poznan, Poland; 7 Department of Neurology and Pediatrics, Medical University of Bialystok, 15-274 Bialystok, Poland; 8 Center of Excellence for Rare and Undiagnosed Disorders, Medical University of Warsaw, 02-091 Warsaw, Poland; 9 Department of Medical Genetics, Medical University of Warsaw, 02-106 Warsaw, Poland; 10 Department and Clinics of Pediatrics, Hematology and Oncology, Medical University of Gdansk, 80-952 Gdansk, Poland

**Keywords:** Klippel–Feil syndrome, Spondylocarpotarsal synostosis syndrome, *FLNB*, Kabuki syndrome, Sacral agenesis with vertebral anomalies, next-generation sequencing, chromosomal microarray analysis, spinal deformities

## Abstract

Congenital vertebral malformations (CVMs), affecting approximately 0.5–1 per 1000 live births, occur either in an isolated form or as part of syndromic disorders. Despite the identification of numerous causative genes for CVMs, the molecular etiology of most cases remains unknown. In this study, we applied a three-tiered diagnostic approach (chromosomal microarray analysis, followed by custom gene panel analysis, and exome/genome sequencing) in a cohort of 34 patients with CVMs. We achieved a 12% diagnostic success rate, identifying a deletion upstream of *SOX9* and pathogenic or likely pathogenic variants in *FLNB* and *KMT2D*. Most pathogenic variants were detected by exome or genome sequencing, while earlier-tier analyses yielded limited results. We also identified two candidate genes, *NSD2* and *TBXT*, that may contribute to the phenotype observed in our patients, but warrant future functional validation. Our work expands the molecular spectrum of CVMs and highlights the utility of comprehensive genomic testing for improving diagnosis and understanding of vertebral development disorders.

## 1. Introduction

Congenital vertebral malformations (CVMs) are a heterogeneous group of skeletal disorders, causing chronic pain and disability. Their estimated prevalence is approximately 1–2 per 2000 live births, although the actual incidence is likely higher due to underdiagnosis. CVMs are classified as segmentation, formation, or mixed defects. The disorder may occur in isolation or as part of syndromes including Klippel–Feil, Alagille, Kabuki, CHARGE, and Spondylocarpotarsal synostosis syndromes [1,2,3,4,5]. Genetic factors explain only 10–20% of cases [6,7,8]. More than 100 genes have been linked to CVMs, most of which encode developmental signaling regulators crucial for spinal morphogenesis [4]. In addition, maternal drug intake and maternal diseases during pregnancy are environmental risk factors for CVMs.

Klippel–Feil syndrome (KFS) is a rare skeletal disorder characterized by the fusion of two or more cervical vertebrae. The classic clinical features of the disease are a short neck, a low posterior hairline, and limited neck motion. Other symptoms include scoliosis, Sprengel’s deformity, urinary or gastrointestinal malformations, congenital heart disease, hearing loss, and neurologic problems [9,10]. Pathogenic variants in *GDF6*, *GDF3*, *MEOX1*, *MYO18B*, and *RIPPLY2* explain a subset of cases of KFS. In the last decade, next-generation (NGS) studies have identified several candidate genes [9,10,11,12,13]. Despite these advances, the etiology of most KFS cases is unresolved.

Although researchers have explained the molecular basis of specific syndromes associated with vertebral defects, the etiology of a substantial proportion of CVMs remains unclear. We investigated 34 patients with CVMs, including a notable subgroup presenting with KFS (*n* = 16). The genetic basis of the disease was examined using a tiered diagnostic strategy. The study represents the first systematic application of this approach in an international CVM cohort.

## 2. Results

### 2.1. Cohort Constitution

We summarized the phenotypic features of the patients in Table 1. We enrolled 34 probands diagnosed with CVM, primarily patients with severe scoliosis or kyphosis. We observed a slight female predominance, with a ratio of approximately 3:2. The affected patients ranged in age from 2 to 41 years. The most common vertebral anomalies were block vertebrae and hemivertebrae. KFS was the most frequent clinical and radiological diagnosis. CVMs occurred more often in the cervical and thoracic regions than in the lumbar, sacral, and coccygeal vertebrae. The most common extra-spinal anomalies were craniofacial dysmorphism (hypertelorism, epicanthus, micrognathia, facial asymmetry, low-set ears, and triangular face). Other consistent clinical features in our cohort included renal abnormalities (horseshoe or fused kidneys, unilateral renal agenesis, and pelvic malposition) and cardiovascular defects (atrial and ventricular septal defects and tricuspid valve insufficiency).

### 2.2. Genetic Results

**Figure 1:**
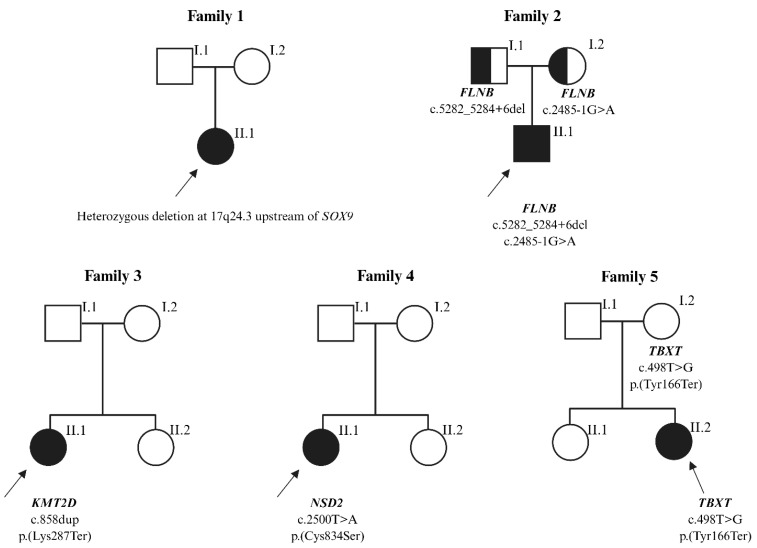
Pedigrees and segregation of the variants identified in patients with congenital vertebral malformations. Filled symbols indicate affected individuals, open symbols indicate unaffected individuals. Half-filled symbols denote heterozygous carriers of the indicated variant. The arrow marks the proband. The *TBXT* variant was considered a candidate finding; therefore, carrier status was annotated next to the symbol and not color-coded.

**Figure 2:**
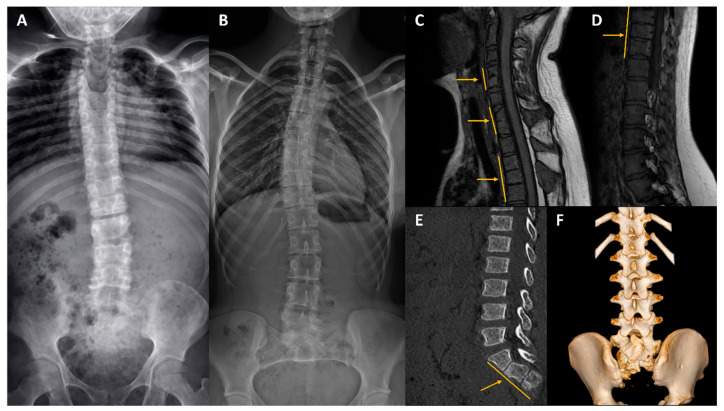
Representative imaging findings of congenital vertebral malformations in patients 2, 4, and 5. Standing anteroposterior spine radiograph, showing spinal deformity with block vertebrae (**A**). Standing anteroposterior radiograph demonstrating scoliosis with vertebral rotation (**B**). Sagittal MRI images showing multilevel congenital block vertebrae (arrows/lines) (**C**,**D**). Sagittal CT reconstruction (**E**) and 3D volume-rendered CT (**F**) demonstrating a malformed, hypoplastic sacrum with only S1 and S2 segments, and partially developed S3 segment, and absent coccyx (arrow/line). (**A**): Patient 2; (**B**–**D**): Patient 4; (**E**,**F**): Patient 5. Detailed radiological characteristics are provided in Table 3.

Our diagnostic success was 12% (*n* = 4). Using array comparative genomic hybridization (aCGH), we detected a *de novo* interstitial deletion at 17q24.3 in a patient with acampomelic campomelic dysplasia. Breakpoint sequencing refined its size to 1.671 Mb (chr17:70,259,128–71,930,429; hg38). The deletion encompassed eleven known *SOX9* upstream regulatory elements [14]. Targeted NGS of a custom 42-gene panel did not allow us to establish a molecular diagnosis in 12 patients with KFS. Consequently, 33 individuals proceeded to the next stage of the diagnostic workflow. Whole-exome sequencing (WES) revealed five heterozygous variants in the subsequent patients: c.2485-1G>A and c.5282_5284+6del in the *FLNB* gene (linked to spondylocarpotarsal synostosis syndrome), c.858dup p.(Lys287Ter) in the *KMT2D* gene (linked to Kabuki syndrome), c.2500T>A p.(Cys834Ser) in the *NSD2* gene (linked to Rauch-Steindl syndrome), and c.498T>G p.(Tyr166Ter) in the *TBXT* gene (linked to sacral agenesis with vertebral anomalies). Among these, only the pathogenic variant in *KMT2D* has been previously reported in the medical literature. We summarized the genetic findings from our cohort in Table 2 and presented the segregation of the variants in Figure 1. The clinical characteristics of variant-positive patients are presented in Table 3, with representative imaging findings shown in Figure 2.

## 3. Discussion

**Figure 3:**
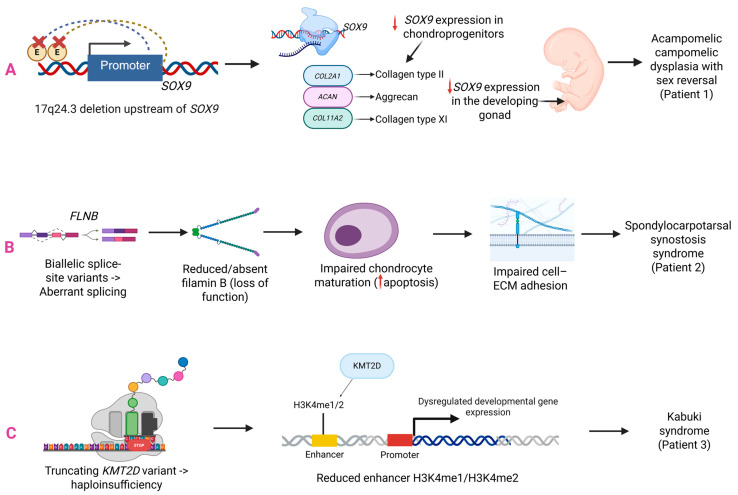
Functional interpretation of disease-causing variants identified in this study. (**A**) A heterozygous 17q24.3 deletion located upstream of *SOX9* is expected to disrupt long-range regulatory control of gene expression, with downstream consequences for chondrogenesis and gonadal development. The molecular characteristics of this case have been described in detail in a previously published report [14]. (**B**) Biallelic splice-site variants in *FLNB* are predicted to result in aberrant splicing and loss of filamin B function. Filamin B is required for proper cytoskeletal organization, chondrocyte maturation, and cell-extracellular matrix interactions, providing a mechanistic explanation for the skeletal phenotype observed in spondylocarpotarsal synostosis syndrome. (**C**) A truncating variant in *KMT2D* leads to haploinsufficiency of a key chromatin regulator involved in enhancer-associated histone methylation. Reduced enhancer activity and dysregulated developmental gene expression lead to clinical features of Kabuki syndrome. Solid arrows indicate the proposed direction of the functional cascade. Dotted curved lines denote putative long-range enhancer-promoter interactions. Red “X” marks deleted regulatory elements. Red downward arrows indicate reduced gene expression. Abbreviations: ECM - extracellular matrix.

The heterogeneity and complexity of the genetic architecture of CVMs pose a challenge to understanding the etiology of the disease. In this study, we reported 34 patients in whom we applied a three-step diagnostic protocol. The genetic analyses revealed a *de novo* 17q24.3 deletion upstream of *SOX9*, a pathogenic variant in *KMT2D*, two likely pathogenic variants in *FLNB*, and novel candidate variants in *NSD2* and *TBXT*. The molecular diagnostic rate was 12%, aligning with yields from large cohort studies [15,16]. A detailed clinical and genetic description of Patient 1 has been published previously [14]. The remaining cases from our cohort are discussed below. WES provided a diagnosis in three patients, representing the most effective diagnostic method in our cohort. In patient 2, WES revealed compound heterozygous likely pathogenic splice-site variants in *FLNB*: c.2485-1G>A and c.5282_5284+6del, both of which were novel. The 2-year-old child exhibited clinical features of spondylocarpotarsal synostosis syndrome (SCT), including short stature, short neck, spinal lordosis, vertebral fusions, and dysmorphic facial features. Hand radiographs obtained at the time of evaluation did not demonstrate carpal synostosis. However, skeletal maturation was markedly delayed, with a left-hand bone age corresponding to 9 months for the long bones and 18 months for the carpal bones. Delayed carpal ossification has been reported in SCT, and carpal synostosis may not be detectable early in childhood, becoming apparent on follow-up [17]. Manifestations observed in fewer than 25% of individuals with SCT include brachydactyly, clinodactyly, clubfoot, cleft palate, and enamel hypoplasia [18,19,20]. The patient had clinodactyly of the fifth fingers but did not present with the other uncommon features. To date, approximately 40 cases of SCT have been documented, with only five patients having compound heterozygous variants in *FLNB* [21,22,23]. Most reported variants included SNVs, small indels, and intragenic deletions that result in nonsense or frameshifting effects [24,25]. Only one splice-site variant has been documented in a patient with SCT [21]. Our proband represents the first case of SCT caused by biallelic canonical splice-site variants in the *FLNB* gene. The detected variants are predicted to disrupt splicing and generate loss-of-function alleles. In two *Flnb*-deficient mouse models, homozygous loss of *Flnb* resulted in growth restriction and a pattern of skeletal abnormalities that resembled the *FLNB*-related recessive phenotype, including delayed endochondral ossification, reduced cartilage matrix, vertebral segmentation defects, vertebral and rib fusions, as well as abnormal spinal curvature (kyphosis/scoliosis). Experimental analyses indicated that loss of *Flnb* affected chondrocyte maturation, leading to increased apoptosis in developing skeletal elements (Figure 3B) [26,27]. Future studies should incorporate RNA sequencing to confirm the splicing defects of the variants and assess their functional consequences.

The second patient with an established molecular diagnosis (P3) carried a *de novo* nonsense variant in *KMT2D* (c.858dup, p.Lys287Ter), which confirmed the diagnosis of Kabuki syndrome (KS; OMIM #147920). Our proband showed the typical features of KS, including distinctive facial appearance, kidney defects, skeletal changes, and mild intellectual disability [28]. The same variant was reported in another individual with KS with a similar clinical picture [29]. Notably, the patient presented with a butterfly vertebra. CVMs have been reported only occasionally in KS and may extend the known skeletal phenotype associated with this condition [30,31]. The *KMT2D* gene encodes a histone methyltransferase involved in enhancer-associated H3K4 methylation. Disruption of this process can alter developmental transcription needed for normal skeletal development (Figure 3C). Studies in mice have shown that *Kmt2d* loss-of-function disrupts osteochondral differentiation and endochondral ossification, and recapitulates craniofacial and growth phenotypes seen in Kabuki syndrome [32,33]. Our case highlights the importance of considering *KMT2D* testing in patients with vertebral and multisystem developmental defects.

**Figure 4:**
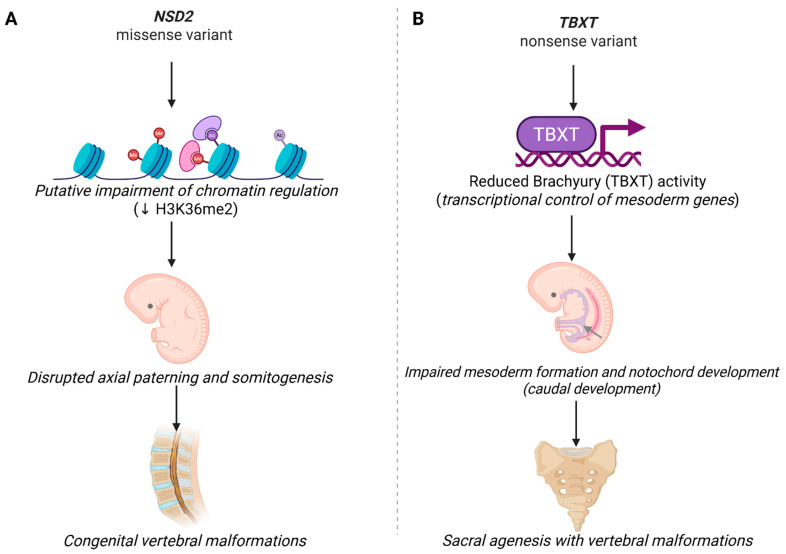
Mechanisms for candidate gene variants identified in this study. (**A**) *NSD2*: putative disruption of chromatin regulation during embryogenesis. (**B**) *TBXT*: reduced Brachyury-mediated transcriptional regulation during caudal axis development. Schemes are conceptual and summarize hypothesized links between gene function and the observed vertebral phenotypes.

In addition to the confirmed disease-causing variants, our analysis identified novel candidate variants for CVMs. WES identified a *de novo* missense variant of uncertain significance (VUS) in *NSD2* (c.2500T>A p.Cys834Ser) in patient 4 and a likely pathogenic variant in *TBXT* (c.498T>G, p.Tyr166Ter) in patient 5. A schematic summary of putative developmental mechanisms for *NSD2* and *TBXT* is provided in Figure 4.

Patient 4 had a clinical phenotype highly concordant with Rauch-Steindl syndrome (RSS), i.e., microcephaly, facial dysmorphisms, intellectual disability, failure to thrive, short stature, and muscular hypotonia. The *NSD2* gene encodes a histone lysine methyltransferase involved in chromatin regulation and orchestrates developmental gene-expression programs. Disruption of chromatin-mediated transcriptional regulation during early embryogenesis may interfere with somitogenesis and, consequently, compromise axial patterning and vertebral morphogenesis. While most pathogenic variants in the *NSD2* gene are truncating, several *de novo* missense variants have been documented in individuals with RSS [34,35]. The strong phenotype concordance between the patient and the RSS phenotype sets, along with the *de novo* origin, suggests that the variant is a potential contributor to the disease. Further studies, including transcriptomic and methylation analyses, will be relevant to confirm our findings.

Patient 5 carried a likely pathogenic nonsense variant in *TBXT* (c.498T>G, p.Tyr166Ter). The variant was also present in a healthy mother of the proband. The *TBXT* gene encodes Brachyury, which plays a central role in mesoderm development and formation of the body axis. Pathogenic, mostly biallelic variants in *TBXT* have been reported in patients with sacral agenesis with vertebral anomalies [36,37,38]. CVMs observed in our patient are consistent with this clinical spectrum, indicating that *TBXT* may play a role in the disease mechanism. Although the disorder follows an autosomal recessive inheritance pattern, the proband carried a heterozygous loss-of-function variant inherited from her healthy mother. Similarly, another study described a heterozygous *TBXT* variant transmitted from an unaffected parent to a child with sacral agenesis [38]. Taking together, we suggest that the variant is more likely a strong predisposing factor than a single causative allele.

We assessed the identified genes using the STRING database. Overall, little direct functional connectivity was observed. A single association was noted between *KMT2D* and *NSD2*, reflecting their shared involvement in chromatin regulation. No broader interaction network was apparent among the remaining genes.

We were unable to obtain a definitive molecular diagnosis for most patients in our cohort, including all individuals with KFS. CVMs may result from complex genetic mechanisms, such as oligogenic, digenic, or polygenic inheritance. In addition, environmental and epigenetic changes can influence disease [4,9,39]. These factors, along with the limitations of current sequencing technologies, may explain why many patients still do not receive a genetic diagnosis. Future analyses should include RNA sequencing, methylation profiling, or long-read sequencing to improve the diagnostic process in unresolved cases.

Female patients were overrepresented in our cohort, including four of the five patients with a molecular finding. However, for *FLNB*, *KMT2D*, *NSD2*, *SOX9*, and *TBXT*, published cases include both sexes, and there is no clear evidence that variant effects on vertebral development are sex-dependent. The present study is underpowered to address sex effects, and this question should be revisited in larger cohorts.

In this study, we provide a combined clinical and molecular characterization of CVMs in a cohort of Polish patients. In addition to known disease-causing variants, we identified pathogenic or candidate variants in *FLNB*, *KMT2D*, *NSD2*, and *TBXT*, as well as a pathogenic deletion upstream of *SOX9*. Further progress in understanding CVM pathogenesis will require studies integrating genomic data with functional and epigenetic analyses.

## 4. Methods

### 4.1. Patient Recruitment and Clinical Evaluation

We enrolled 34 Polish patients with CVMs, who underwent physical examination and spinal imaging (radiography, computed tomography, and magnetic resonance imaging). Isolated and syndromic cases were eligible, including structural CVMs (e.g., hemivertebrae, butterfly vertebrae, and vertebral fusions) and KFS. We excluded patients with acquired vertebral deformities (post-traumatic, infectious, or neoplastic) as well as individuals with incomplete clinical data, no DNA available for testing, or a lack of written informed consent. We reviewed each participant’s medical records to define the vertebral malformation phenotype and identify any associated anomalies involving the spinal cord, heart, kidneys, brain, or other skeletal structures. This study was approved by the Institutional Review Board of the Poznan University of Medical Sciences ethics committee. Written informed consent for participation and publishing the information and images was obtained from all patients and the parents of underage participants before genetic testing.

### 4.2. Genetic Analyses

**Figure 5:**
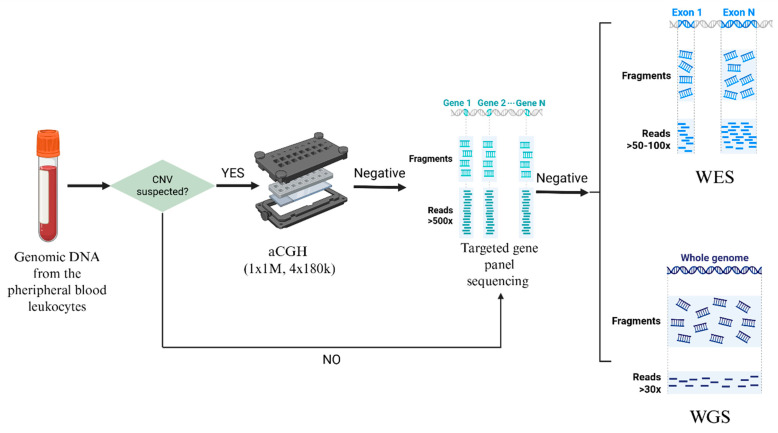
The diagnostic workflow implemented in our study for 34 patients affected with congenital vertebral malformations. Genetic testing comprised array comparative genomic hybridization (aCGH) (*n* = 17), targeted gene panel sequencing (*n* = 12), whole-exome sequencing (WES) (*n* = 9), and whole-genome sequencing (WGS) (*n* = 25).

We extracted genomic DNA (gDNA) from peripheral blood leukocytes using the MagCore HF16 Automated Nucleic Acid Extractor (RBC Bioscience Corp., New Taipei City, Taiwan). The diagnostic workflow was implemented in a stepwise manner. First, aCGH was performed. Next, we applied targeted NGS of a custom gene panel to patients diagnosed with KFS. Finally, WES or whole-genome sequencing (WGS) was performed in cases that remained negative after the preceding analyses. All candidate genetic variants were further confirmed by segregation analysis using Sanger sequencing. Figure 5 depicts this study design and diagnostic workflow.

#### 4.2.1. aCGH

Array comparative genomic hybridization (aCGH) was performed in patients with a clinical suspicion of pathogenic copy number variations (CNVs). Analyses were conducted using the SurePrint G3 Human CGH Microarray 1 × 1 M or 4 × 180 k platform (Agilent Technologies, Santa Clara, CA, USA) with a median probe spacing of 2.1 kb, according to the manufacturer’s instructions. Hybridization signals were recorded with the SureScan Dx Microarray Scanner (Agilent Technologies) and analyzed in Agilent CytoGenomics software (v5.0.2.5). The interpretation of CNVs drew on multiple reference resources, including DECIPHER, the Database of Genomic Variants (DGV), and Mouse Genome Informatics (MGI). Pathogenic CNVs were confirmed by quantitative PCR (qPCR) and evaluated for parental segregation. The qPCR procedure was performed as previously described [14].

#### 4.2.2. Targeted NGS Panel

A cohort with KFS with negative aCGH results underwent targeted NGS. We designed a custom panel comprising 42 genes linked to CVMs using the Ion AmpliSeq Designer platform (v7.8.7; Thermo Fisher Scientific, Waltham, MA, USA) (Supplementary Table S1). We selected genes based on a review of the medical literature and their documented association with CVMs in genetic databases (OMIM and ClinVar). Libraries were prepared from 50 ng gDNA using the Ion AmpliSeq™ Library Kit 2.0 (Thermo Fisher Scientific, Waltham, MA, USA) and sequenced on the Ion Torrent S5 platform. The detailed laboratory workflow has been previously described [40]. Sequencing reads were processed using Torrent Suite v5.20.8.0 to perform base calling, quality control, and alignment to the GRCh37/hg19 reference genome. Variants were filtered using the following thresholds: read depth ≥ 20, PHRED quality ≥ 40, and allele frequency ≥ 0.15. NGS alignments were visualized using the Integrative Genomics Viewer (version 2.19.1; Broad Institute and the Regents of the University of California). For variant prioritization and interpretation, we evaluated the variants using reference databases, including HGMD, ClinVar, dbSNP, and gnomAD. The in silico prediction tools used in our analysis included SIFT, Polyphen-2, CADD, and REVEL. The variant’s pathogenicity was interpreted in accordance with the American College of Medical Genetics (ACMG) guidelines.

#### 4.2.3. WES and WGS

WES or WGS was performed in certified diagnostic laboratories on DNA isolated from the patient’s blood samples. In WES, the coding regions and adjacent intronic sequences were enriched using a custom in-solution hybridization kit (Twist Bioscience, San Francisco, USA), achieving an average depth of approximately 50×. WGS libraries were prepared with the TruSeq DNA Nano Kit (Illumina, San Diego, CA, USA), yielding a mean depth of 30×. Prepared libraries were sequenced on the Illumina NovaSeq platform (Illumina, San Diego, USA). Sequencing reads were demultiplexed with bcl2fastq2, and adapters were removed using Skewer (v0.2.2). The reads were aligned to the GRCh38/hg38 human reference genome. PCR duplicates and low-quality reads were filtered out, and variant calling was performed using in-house bioinformatics software. CNV calling was performed for WGS as part of the diagnostic bioinformatics workflow, whereas WES analysis did not include CNV calling. First, we examined rare variants with a minor allele frequency (MAF) < 1%. In addition, pathogenic, likely pathogenic, or variants of uncertain significance in genes linked to CVMs were examined. Downstream analyses followed the workflow described for the targeted NGS panel.

#### 4.2.4. Sanger Sequencing

We have analyzed the genomic region of interest using Sanger sequencing to confirm the presence of each pathogenic, likely pathogenic, and uncertain variant detected in NGS-based analyses. Primers were designed using the Primer3 tool version 0.4.0. PCR amplification and purification were performed according to standard laboratory protocols. Sanger sequencing was performed with dye-terminator chemistry (kit v.3, ABI 3130XL) and run on Applied Biosystems Prism 3700 DNA Analyzer.

## Figures and Tables

**Table 1:** Clinical characteristics of the cohort. CVMs—congenital vertebral malformations.

Characteristics	Cohort with CVMs (*n*, %)
**Sex**	
**Female**	21 (62%)
Male	13 (38%)
**CVMs**	
Failure of formation	10 (29%)
**Failure of segmentation**	12 (35%)
Mixed	13 (38%)
**Forms of CVMs**	
Syndromes	
Klippel–Feil syndrome	21 (62%)
Jarcho–Levin syndrome	16 (47%)
Campomelic dysplasia	1 (3%)
Sacral agenesis with vertebral anomalies	1 (3%)
Kabuki syndrome	1 (3%)
**Spondylocarpotarsal synostosis syndrome**	1 (3%)
Isolated CVMs	13 (38%)
**Location of CVMs**	
Cervical	27 (79%)
Thoracic	24 (71%)
Lumbar	9 (26%)
Sacral	4 (12%)
Coccygeal	1 (3%)
**Associated anomalies**	
Cardiovascular disorders	8 (24%)
Facial dysmorphism	12 (35%)
**Gastrointestinal anomalies**	5 (14%)
Hearing loss	4 (12%)
Limbs	6 (18%)
Neurological anomalies	7 (21%)
Renal abnormalities	9 (26%)
Rib malformations	15 (44%)
Vision impairment	6 (18%)

**Table 2:** Genetic findings in the study cohort. Online tools used: Varsome Premium. Abbreviations: aCGH—array comparative genomic hybridization, ACMG—American College of Medical Genetics and Genomics, ND—not determined, VUS—variant of uncertain significance, WES—whole exome sequencing, ^a^ SpliceAI predicted loss of the canonical splice acceptor site for FLNB c.2485-1G>A (acceptor loss Δ = 0.98), ^b^ SpliceAI predicted loss of the splice donor site for FLNB c.5282_5284+6del (donor loss Δ = 1.00).

Patient	Gene	Transcript	Variant	Zygosity	Inheritance	gnomAD Exomes (v4)	ACMG Variant Classification	Criteria	Molecular Method
P1	*SOX9* regulatory region	N/A	NC_000017.11:g. 70259128_71930429del	Het	de novo	N/A	Pathogenic	ND	aCGH +breakpoint mapping
P2	*FLNB*	NM_001457.4	c.2485-1G>A ^a^ c.5282_5284+6del ^b^	Het Het	Maternal Paternal	0 0.000000684	Likely pathogenic Likely pathogenic	PVS1 Very Strong PM2 Supporting PVS1 Very Strong PM2 Supporting	WES
P3	*KMT2D*	NM_003482.4	c.858dup p.(Lys287Ter)	Het	de novo	0	Pathogenic	PS4 Moderate PVS1 Very Strong PM2 Moderate PS2 Strong	WES
P4	*NSD2*	NM_001042424.3	c.2500T>A p.(Cys834Ser)	Het	de novo	0	VUS	PP3 Strong PP2 Supporting PM2 Supporting PS2 Strong BP1 Supporting	WES
P5	*TBXT*	NM_001366285.2	c.498T>G p.(Tyr166Ter)	Het	Maternal	0	Likely pathogenic	PVS1 Very Strong PM2 Moderate	WES

**Table 3:** Summary of clinical features of the patients with detected genetic alterations. *—A detailed description is provided in a separate publication [14].

Patient	Sex	Age at Admission	Syndrome	Skeletal Anomalies	Other Malformations
P1	F	2	Acampomelic campomelic dysplasia	Severe thoracic kyphoscoliosis, reduced vertebral height, rotation, canal stenosis, bell-shaped thorax, hypoplastic scapulae, narrow iliac wings, short neck *	Facial dysmorphism, cleft palate, widely spaced nipples, a sandal gap, 46, XY complete gonadal dysgenesis *
P2	M	2	Spondylocarpotarsal synostosis syndrome	Right-sided torticollis, bony block of the vertebral arches from Th5-Th12 and L2-S1, the thoracic and lumbar lordosis, lumbar kyphosis, short neck, fifth finger clinodactyly	Short stature, psychomotor developmental delay, facial dysmorphia (dolichocephaly, frontal prominence, micrognathia, epicanthal folds, facial asymmetry), limited tongue protrusion, dilated renal pelvis, cerebellar hypoplasia
P3	F	2	Kabuki syndrome	Butterfly vertebrae at Th6 and Th12, genu valgum, left hip dysplasia	Facial dysmorphia (hypertelorism, broad nasal bridge, high forehead, retrognathia), psychomotor developmental delay, speech delay, duplex kidney, horseshoe kidney, joint laxity
P4	F	13	Rauch-Steindl syndrome	Scoliosis with vertebral rotation, cervical and lumbar lordosis with thoracic kyphosis, bony blocks at C4-C5, C7-Th1, Th3-Th5, Th8-Th10, bilateral cervical ribs, fifth finger clinodactyly	Short stature, postnatal growth retardation, failure to thrive, small head circumference, microcephaly, facial dysmorphism (short philtrum, prominent glabella, hypertelorism, low-set ears, posteriorly rotated ears, arched eyebrows, wide nasal bridge, thin lips, thin downturned corners of the mouth), astigmatism, craniofacial asymmetry, atrial septal defect type II, dental abnormalities, developmental delay, delayed walking, hypotonia, impaired intellectual development
P5	F	2	Sacral agenesis with vertebral anomalies	Hypoplastic sacrum with only segments S1–S3 present (S1–S2 hypoplastic, S3 partially formed), agenesis of the coccyx, congenital hip dysplasia, bilateral clubfoot	Spinal cord malformations (absent conus medullaris), right ectopic pelvic kidney with hypoplasia, dysmorphic features (abnormal gluteal crease, abnormal cutaneous sinus tract)

## Data Availability

The original contributions presented in this study are included in the article. Further inquiries can be directed to the corresponding author.

## Supplementary Materials

The following supporting information can be downloaded at: https://www.mdpi.com/article/10.3390/ijms27041752/s1.

## References

[1] Chen Y., Liu Z., Chen J., Zuo Y., Liu S., Chen W., Liu G., Qiu G., Giampietro P.F., Wu N. (2016). The genetic landscape and clinical implications of vertebral anomalies in VACTERL association. J. Med. Genet..

[2] Erol B., Tracy M.R., Dormans J.P., Zackai E.H., Maisenbacher M.K., O’Brien M.L., Turnpenny P.D., Kusumi K. (2004). Congenital scoliosis and vertebral malformations: Characterization of segmental defects for genetic analysis. J. Pediatr. Orthop..

[3] Giampietro P.F., Dunwoodie S.L., Kusumi K., Pourquié O., Tassy O., Offiah A.C., Cornier A.S., Alman B.A., Blank R.D., Raggio C.L. (2009). Progress in the understanding of the genetic etiology of vertebral segmentation disorders in humans. Ann. N. Y. Acad. Sci..

[4] Szoszkiewicz A., Bukowska-Olech E., Jamsheer A. (2024). Molecular landscape of congenital vertebral malformations: Recent discoveries and future directions. Orphanet J. Rare Dis..

[5] Turnpenny P.D., Alman B., Cornier A.S., Giampietro P.F., Offiah A., Tassy O., Pourquié O., Kusumi K., Dunwoodie S. (2007). Abnormal vertebral segmentation and the notch signaling pathway in man. Dev. Dyn..

[6] Liu J., Wu N., Yang N., Takeda K., Chen W., Li W., Du R., Liu S., Zhou Y., Zhang L. (2019). TBX6-associated congenital scoliosis (TACS) as a clinically distinguishable subtype of congenital scoliosis: Further evidence supporting the compound inheritance and TBX6 gene dosage model. Genet. Med..

[7] Wu N., Ming X., Xiao J., Wu Z., Chen X., Shinawi M., Shen Y., Yu G., Liu J., Xie H. (2015). TBX6 null variants and a common hypomorphic allele in congenital scoliosis. N. Engl. J. Med..

[8] Zhao S., Zhang Y., Chen W., Li W., Wang S., Wang L., Zhao Y., Lin M., Ye Y., Lin J. (2021). Diagnostic yield and clinical impact of exome sequencing in early-onset scoliosis (EOS). J. Med. Genet..

[9] Li Z., Zhao S., Cai S., Zhang Y., Wang L., Niu Y., Li X., Hu J., Chen J., Wang S. (2020). The mutational burden and oligogenic inheritance in Klippel-Feil syndrome. BMC Musculoskelet. Disord..

[10] Saker E., Loukas M., Oskouian R.J., Tubbs R.S. (2016). The intriguing history of vertebral fusion anomalies: The Klippel-Feil syndrome. ChNS.

[11] Alazami A.M., Kentab A.Y., Faqeih E., Mohamed J.Y., Alkhalidi H., Hijazi H., Alkuraya F.S. (2015). A novel syndrome of Klippel-Feil anomaly, myopathy, and characteristic facies is linked to a null mutation in MYO18B. J. Med. Genet..

[12] Karaca E., Yuregir O.O., Bozdogan S.T., Aslan H., Pehlivan D., Jhangiani S.N., Akdemir Z.C., Gambin T., Bayram Y., Atik M.M. (2015). Rare variants in the notch signaling pathway describe a novel type of autosomal recessive Klippel-Feil syndrome. Am. J. Med. Genet. A.

[13] Li Z.Q., Geng M.Z., Zhao S., Wu Z.H., Zhang J.G., Wu N., Wang Y.P. (2021). Clinical Characteristics and Genetic Analysis of Klippel-Feil Syndrome. Acta Acad. Med. Sin..

[14] Szoszkiewicz A., Bukowska-Olech E., Kurzawa P., Sowińska-Seidler A., Niedziela M., Kolesińska Z., Jamsheer A. (2025). Upstream SOX9 deletion in a 46,XY girl with acampomelic campomelic dysplasia and absent minipuberty. Orphanet J. Rare Dis..

[15] Beauregard-Lacroix E., Tardif J., Camurri M.V., Lemyre E., Barchi S., Parent S., Campeau P.M. (2017). Retrospective Analysis of Congenital Scoliosis: Associated Anomalies and Genetic Diagnoses. Spine.

[16] Zhao S., Zhao H., Zhao L., Cheng X., Zheng Z., Wu M., Wen W., Wang S., Zhou Z., Xie H. (2024). Unraveling the genetic architecture of congenital vertebral malformation with reference to the developing spine. Nat. Commun..

[17] Mitter D., Krakow D., Farrington-Rock C., Meinecke P. (2008). Expanded clinical spectrum of spondylocarpotarsal synostosis syndrome and possible manifestation in a heterozygous father. Am. J. Med. Genet. A.

[18] Coêlho K.E., Ramos E.S., Felix T.M., Martelli L., de Pina-Neto J.M., Niikawa N. (1998). Three new cases of spondylocarpotarsal synostosis syndrome: Clinical and radiographic studies. Am. J. Med. Genet..

[19] Isidor B., Cormier-Daire V., Le Merrer M., Lefrancois T., Hamel A., Le Caignec C., David A., Jacquemont S. (2008). Autosomal dominant spondylocarpotarsal synostosis syndrome: Phenotypic homogeneity and genetic heterogeneity. Am. J. Med. Genet. A.

[20] Yang C.-F., Wang C.-H., Siong H’ng W., Chang C.-P., Lin W.-D., Chen Y.-T., Wu J.-Y., Tsai F.-J. (2017). Filamin B Loss-of-Function Mutation in Dimerization Domain Causes Autosomal-Recessive Spondylocarpotarsal Synostosis Syndrome with Rib Anomalies. Hum. Mutat..

[21] Farrington-Rock C., Kirilova V., Dillard-Telm L., Borowsky A.D., Chalk S., Rock M.J., Cohn D.H., Krakow D. (2008). Disruption of the Flnb gene in mice phenocopies the human disease spondylocarpotarsal synostosis syndrome. Hum. Mol. Genet..

[22] Krakow D., Robertson S.P., King L.M., Morgan T., Sebald E.T., Bertolotto C., Wachsmann-Hogiu S., Acuna D., Shapiro S.S., Takafuta T. (2004). Mutations in the gene encoding filamin B disrupt vertebral segmentation, joint formation and skeletogenesis. Nat. Genet..

[23] Ramos-Mejía R., Del Pino M., Aza-Carmona M., Abbate S., Obregon M.G., Heath K.E., Fano V. (2024). Novel FLNB Variants in Seven Argentinian Cases with Spondylocarpotarsal Synostosis Syndrome. J. Pediatr. Genet..

[24] Fukushima K., Parthasarathy P., Wade E.M., Morgan T., Gowrishankar K., Markie D.M., Robertson S.P. (2021). Intragenic Deletions in FLNB Are Part of the Mutational Spectrum Causing Spondylocarpotarsal Synostosis Syndrome. Genes.

[25] Yasin S., Makitie O., Naz S. (2021). Spondylocarpotarsal synostosis syndrome due to a novel loss of function FLNB variant: A case report. BMC Musculoskelet. Disord..

[26] Lu J., Lian G., Lenkinski R., De Grand A., Vaid R.R., Bryce T., Stasenko M., Boskey A., Walsh C., Sheen V. (2007). Filamin B mutations cause chondrocyte defects in skeletal development. Hum. Mol. Genet..

[27] Zhou X., Tian F., Sandzén J., Cao R., Flaberg E., Szekely L., Cao Y., Ohlsson C., Bergo M.O., Borén J. (2007). Filamin B deficiency in mice results in skeletal malformations and impaired microvascular development. Proc. Natl. Acad. Sci. USA.

[28] Niikawa N., Matsuura N., Fukushima Y., Ohsawa T., Kajii T. (1981). Kabuki make-up syndrome: A syndrome of mental retardation, unusual facies, large and protruding ears, and postnatal growth deficiency. J. Pediatr..

[29] Fan H., Wang Y., Wu Y., Jia L., Wang L., Shen Y. (2024). Clinical and genetic characteristics of four children with Kabuki syndrome due to de novo variants of KMT2D gene. Chin. J. Med. Genet..

[30] Di Candia F., Fontana P., Paglia P., Falco M., Rosano C., Piscopo C., Cappuccio G., Siano M.A., De Brasi D., Mandato C. (2022). Clinical heterogeneity of Kabuki syndrome in a cohort of Italian patients and review of the literature. Eur. J. Pediatr..

[31] White S.M., Thompson E.M., Kidd A., Savarirayan R., Turner A., Amor D., Delatycki M.B., Fahey M., Baxendale A., White S. (2004). Growth, behavior, and clinical findings in 27 patients with Kabuki (Niikawa-Kuroki) syndrome. Am. J. Med. Genet. A.

[32] Gao C.W., Lin W., Riddle R.C., Chopra S., Kim J., Boukas L., Hansen K.D., Björnsson H.T., Fahrner J.A. (2024). Growth deficiency in a mouse model of Kabuki syndrome 2 bears mechanistic similarities to Kabuki syndrome 1. PLoS Genet..

[33] Shpargel K.B., Mangini C.L., Xie G., Ge K., Magnuson T. (2020). The KMT2D Kabuki syndrome histone methylase controls neural crest cell differentiation and facial morphology. Development.

[34] Zanoni P., Steindl K., Sengupta D., Joset P., Bahr A., Sticht H., Lang-Muritano M., van Ravenswaaij-Arts C.M.A., Shinawi M., Andrews M. (2021). Loss-of-function and missense variants in NSD2 cause decreased methylation activity and are associated with a distinct developmental phenotype. Genet. Med..

[35] Nishi E., Yanagi K., Kaname T., Okamoto N. (2024). Clinical details of individuals with Rauch-Steindl syndrome due to NSD2 truncating variants. Mol. Genet. Genom. Med..

[36] Chen S., Lei Y., Yang Y., Liu C., Kuang L., Jin L., Finnell R.H., Yang X., Wang H. (2024). A mutation in TBXT causes congenital vertebral malformations in humans and mice. J. Genet. Genom..

[37] Postma A.V., Alders M., Sylva M., Bilardo C.M., Pajkrt E., van Rijn R.R., Schulte-Merker S., Bulk S., Stefanovic S., Ilgun A. (2014). Mutations in the T (brachyury) gene cause a novel syndrome consisting of sacral agenesis, abnormal ossification of the vertebral bodies and a persistent notochordal canal. J. Med. Genet..

[38] Ghebranious N., Blank R.D., Raggio C.L., Staubli J., McPherson E., Ivacic L., Rasmussen K., Jacobsen F.S., Faciszewski T., Burmester J.K. (2008). A missense T (Brachyury) mutation contributes to vertebral malformations. J. Bone Miner. Res..

[39] Yang N., Wu N., Zhang L., Zhao Y., Liu J., Liang X., Ren X., Li W., Chen W., Dong S. (2019). TBX6 compound inheritance leads to congenital vertebral malformations in humans and mice. Hum. Mol. Genet..

[40] Bukowska-Olech E., Popiel D., Koczyk G., Sowińska-Seidler A., Socha M., Wojciechowicz B., Dawidziuk A., Larysz D., Jamsheer A. (2020). Adapting SureSelect enrichment protocol to the Ion Torrent S5 platform in molecular diagnostics of craniosynostosis. Sci. Rep..

